# The assessment of internal adaptation and fracture resistance of glass ionomer and resin-based restorative materials applied after different caries removal techniques in primary teeth: an *in-vitro* study

**DOI:** 10.7717/peerj.14825

**Published:** 2023-03-28

**Authors:** Akif Demirel, Ayşe Işıl Orhan, Arda Büyüksungur

**Affiliations:** 1 Faculty of Dentistry, Pediatric Dentistry Department, Ankara University, Ankara, Yenimahalle, Turkey; 2 Faculty of Dentistry, Pediatric Dentistry Department, Ankara Yıldırım Beyazıt University, Ankara, Turkey; 3 Faculty of Dentistry, Department of Basic Medical Sciences, Ankara University, Ankara, Yenimahalle, Turkey

**Keywords:** Compomer, Glass ionomer, Firm dentin, Primary teeth, Selective caries removal

## Abstract

**Background:**

The aim of this study was to evaluate the 3-dimensional (3D) internal adaptation (IA) and fracture resistance (FR) of compomer and glass ionomers applied after conventional caries removal to sound dentin (CCRSD) and selective caries removal to firm dentin (SCRFD) in *in-vitro*.

**Methods:**

Thirty extracted primary molars were randomly assigned to three main groups (*n* = 10) as glass hybrid restorative (GHR) (Equia Forte^®^ HT), conventional glass ionomer (CGIR) (Voco Ionofil Molar) and compomer (Dyract XP). Each group was randomly divided into two subgroups according to caries removal technique as CCRSD (*n* = 5) and SCRFD (*n* = 5). The restoration procedures were completed after caries removal (CCRSD or SCRFD) in all samples. Then, specimens were subjected to IA and FR tests. Data were analyzed with Student’s t, one-way ANOVA, and Kruskal Wallis-H tests. The correlation between IA and FR results was analyzed with a Pearson test. The statistical significance level was considered as 5%.

**Results:**

While CCRSD showed superior IA results than SCRFD for all restorative materials (*p* < 0.05), no statistical difference was found between CCRSD and SCRFD in FR assessment (*p* > 0.05). In CCRSD, compomer showed superior results for IA and FR than glass ionomers (*p* < 0.05). In SCRFD, it was found no significant difference between the restoratives for IA (*p* > 0.05). However, compomer showed superior FR results than glass ionomers (*p* < 0.05). There was moderate negative correlation between internal voids and FR without statistically significant difference (r = −0.333, *p* = 0.072).

**Conclusions:**

Despite the advantages of SCRFD, it was found to be less superior than CCRSD in IA assessment. Therefore, when SCRFD is preferred, a peripheral seal should be provided for ideal restorative treatment. On the other hand, compomer mostly showed superior results compared to others.

## Introduction

Dental caries affects children in terms of general health, well-being and intellectual development. In this sense, the rehabilitation of dental caries in childhood also restores the health and function of the child (Finucane, 2012; Finucane, 2019). From past to present, many approaches and restorative materials have been used for the rehabilitation of caries (Finucane, 2019). However, in recent years, minimum intervention dentistry (MID) has become a popular approach which has been adopted to manage caries lesions (Walsh & Brostek, 2013). Complete caries removal, also known as non-selective caries removal, refers to the complete removal of contaminated (infected) and demineralized (affected) dentin. However, the complete removal of caries is unnecessary when the carious lesion is sealed hermetically, as demineralized dentin has the potential to remineralize, as suggested by the MID philosophy (Ngo et al., 2006; Frencken et al., 2012; Kher & Rao, 2019). In this context, in management of moderately deep carious lesions, selective carious removal to the firm dentin (SCRFD) is recommended (Innes et al., 2016; Schwendicke et al., 2016; Kher & Rao, 2019). SCRFD leaves firm dentin pulpally and the peripheral surfaces of the lesion are removed to hard dentin to provide a peripheral seal zone (Schwendicke et al., 2016). It has been reported that this approach provides the maintenance of the tooth by preventing pulpal exposure and thus keeping vitality of the pulp. However, for this technique to be applicable, the caries lesion should extend to less than the pulpal third of dentin with no periapical pathology in radiographic examination. Additionally, the tooth should show no clinical symptoms indicating pulp inflammation such as spontaneous, severe or persistent pain (Innes et al., 2016; Schwendicke et al., 2016; Kher & Rao, 2019).

It is known that severe dental caries cause decrease in quality of life (Fernandes et al., 2017). Therefore, restorative treatment procedures are essential in management of dental caries in pediatric dentistry. Various restorative materials are used in pediatric dentistry, and the most preferred ones are glass ionomer and resin-based materials (Qvist et al., 2004; Ricketts et al., 2013; Dhar et al., 2015; Ehlers et al., 2019; Rodrigues et al., 2019; Kher & Rao, 2019). Resin-based materials are preferred because of their acceptable aesthetic properties and adhesion to dental tissues. Compomers are light cured restorative materials which show common features with composites and glass ionomers. Therefore, compomers are frequently used in restorations of primary teeth due to their simple handling properties (Krämer & Frankenberger, 2007; Shetty & Shetty, 2012; Ehlers et al., 2019; Finucane, 2019).

Glass ionomer restoratives are preferred due to their fluoride release, chemical adhesion to dental tissues and anticariogenic properties. However, conventional glass ionomer restoratives (CGIR) have some disadvantages such as sensitivity to moisture, low fracture and wear resistance. Mentioned situations have been shown to negatively affect the long-term clinical success of CGIR in Class II restorations (Chadwick & Evans, 2007; Bayrak et al., 2017; Finucane, 2019; Kisby, 2021; Uchimura et al., 2021). For this reason, recently, high viscosity reinforced forms of glass ionomer materials have been introduced to the market. In this context, glass hybrid restorative systems have been developed to improve the inadequate physical properties of conventional glass ionomer systems. Glass hybrid restoratives (GHR) contain smaller and more reactive silicate particles and higher molecular weight acrylic acid and they are frequently preferred in pediatric dentistry due to their strengthened biomechanics (Šalinović et al., 2019; Brkanović et al., 2021).

Different tests are used to measure the *in-vitro* adequacy of restorative materials. The internal adaptation (IA) is an important factor that determine the performance of the restorations. The lack of adaptation at the tooth and restorative material interface is a factor affecting the success and longevity of dental restoratives. Therefore, the interface between the restorative material and enamel/dentin has been a concern, as failure to a reach an ideal seal will result in an unsucessful treatment (Murray et al., 2002; Oguz et al., 2021; Al Tuwirqi et al., 2021). It is challenging to develop restorative materials that do not show lack of adaptation. To assess the restoratives, further studies regarding internal adaptation assessment will be very considerable (Kim & Park, 2014). Internal voids or IA can not be diagnosed with conventional radiographic methods. The evaluation of IA of tooth-restoration interface has generally been performed by dye penetration and by sectioning the tooth samples (Alani & Toh, 1997). In order to evaluate the quality of internal adaptation, the current methods such as optical coherence tomography, scanning electron microscopy and micro computed tomography (Micro-CT) are used. These methods allow to perform qualitative and quantitative assessments in three-dimension at the interface between tooth surfaces and restorative materials (Gjorgievska et al., 2008; Rengo et al., 2015; Han & Park, 2017; Abdelaziz et al., 2020; Al Tuwirqi et al., 2021; Demirel et al., 2021; Mendonça et al., 2021). On the other hand, one of the criteria for evaluating the success of a restorative material is the fracture resistance (FR) test (Virmani, Tandon & Rao, 1997; Seraj et al., 2015; Yildiz, Simsek & Pamir, 2016). However, the limited information is available in dental literature on the effect of lack of IA on FR of restorative materials.

Based on the above-mentioned information, this study aimed to evaluate IA and FR values of the various restorative materials applied after conventional total caries removal and selective removal of carious tissue to firm dentin under *in-vitro* conditions. The secondary goal of this study was to evaluate how the lack of IA affects FR by investigating the correlation level of these two *in-vitro* test methods independent of restoratives and caries removal techniques.

## Materials and Methods

### Ethical approval

This study was approved by the Local Ethics Committee of Ankara University, Faculty of Dentistry (approval number: 16/13). Also, the present research has complied with the Checklist for Reporting *In-Vitro* Studies (CRIS) guidelines discussed for *in-vitro* studies (Krithikadatta, Gopikrishna & Datta, 2014). All the procedures of the present study were conducted in accordance with the principles of Declaration of Helsinki. The study protocol was explained in detail to the parents and the children whose extracted primary teeth were used in this study. Also, the informed consent forms were signed by the parents.

### Sample size determination and power calculation

To analyze the differences between the study groups, a power calculation (effect size (f) = 0.7) indicated that a minimum of 30 sample were required to detect a significant difference (90% power and 5% type I error).

### Inclusion criteria, study design and specimen preparation

A total of 30 primary molar teeth were included in this study after radiological selection. The including criteria for this study were in the following: (i) teeth with moderately deep approximal dentin caries, (ii) teeth without pulpal exposure and (iii) teeth without restoration. In accordance with the definition given by one study (Schwendicke et al., 2016), teeth with moderately deep cavitated dentinal lesions (radiographically, lesions extending less than the pulpal third or quarter of dentin) were included in the study. For this reason, standardized periapical radiographs were used to determine the depth of the caries lesion. Samples conforming to the definition of “moderately deep cavitated dentinal lesions” according to the radiographic examination were included in the study. A total of 30 freshly extracted primary molars meeting these criteria obtained from 25 pediatric dental patients (aged 3–7 years) were used in this study. Primary molar teeth that already required surgical extraction were used for the study procedures and the main reasons for extraction were physiological root resorption of primary molars exceeding 2/3 or orthodontic reasons. In addition, teeth with pulpal exposure during the caries removal procedure were excluded from the study protocol. The samples were stored in 0.9% physiological saline solution until the initiation of the study procedures at room temperature.

**Figure 1:**
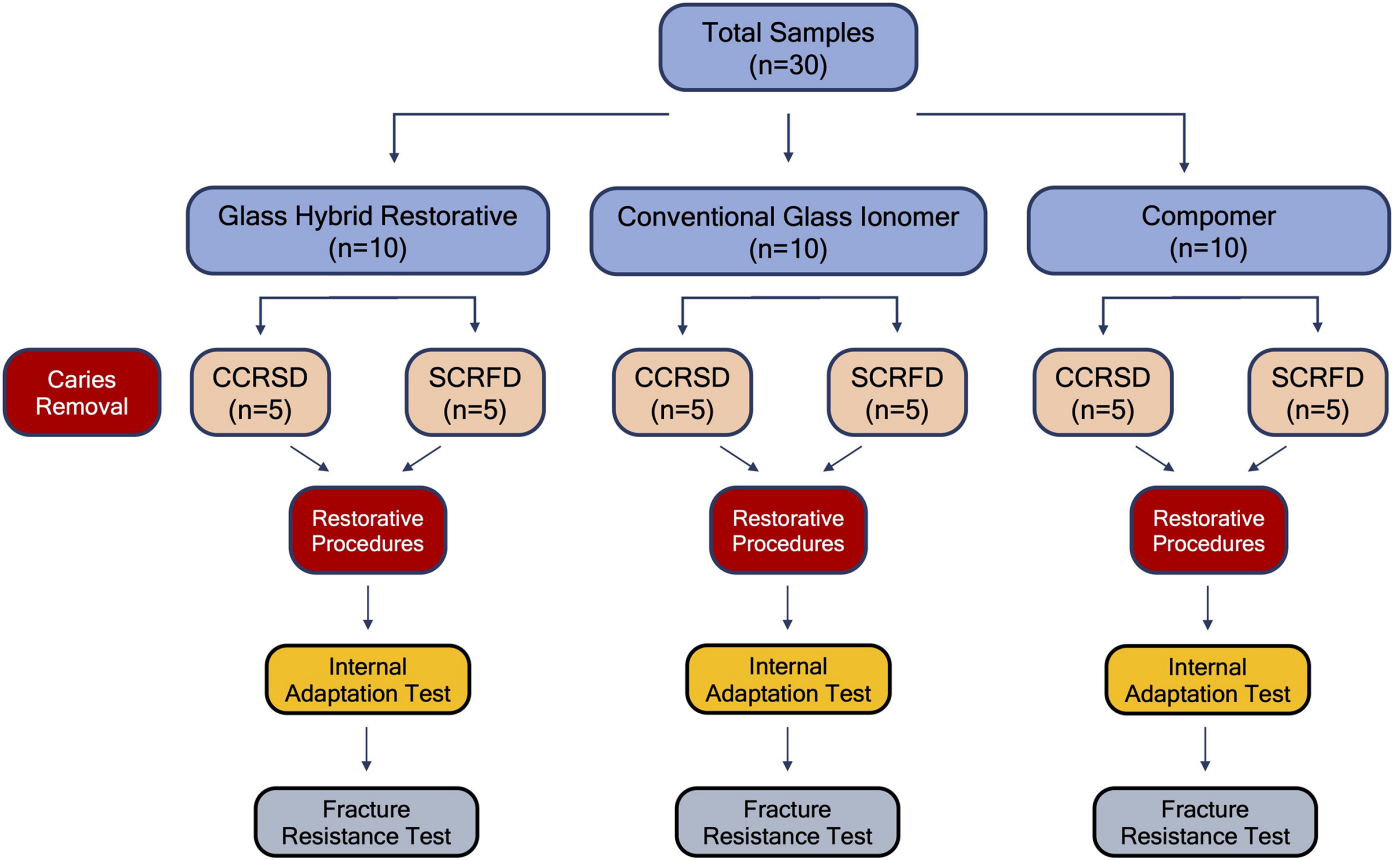
Definition of the study groups and procedures. The flow chart demonstrating the allocation of study groups and the study procedures.

The samples used in this study were randomly assigned to three main groups to be restored with different restorative materials (*n* = 10). These groups were randomly divided into two subgroups as conventional caries removal to sound (hard) dentin (CCRSD) (*n* = 5) and selective caries removal to firm dentin (SCRFD) (*n* = 5) (Fig. 1). For randomization, all the teeth were marked with the symbol of A, B and C (first randomization). Of the randomly selected teeth, those marked with A were assigned to Group 1 (GHR), those marked with B were assigned to Group 2 (CGIR), and those marked with C were assigned to Group 3 (compomer). Also, for each restorative material, half of the 10 teeth (*n* = 5) in each main group were marked with the symbol of 1 and the other half with the symbol of 2 (second randomization). Of the randomly selected teeth, those marked with 1 were assigned to CCRSD, those marked with 2 were assigned to SCRFD. Caries removal and restorative procedures of all specimens were performed by a pediatric dentist (A.D.) with at least 5 years of clinical experience to eliminate calibration problems among different operators. Since the depth of caries lesions in the teeth used in this study were selected to be moderate level, the enamel structure above the caries lesion was removed for all the teeth in all groups with a slow rotating tungsten carbide bur to make the caries visible. In this manner, the caries lesion was made visible and the caries tissue was ready to be removed for both groups (conventional or selective). Subsequently, caries in the samples in CCRSD subgroup was removed up to sound dentin by using round shaped tungsten carbide burs with slow handpiece. On the other hand, in the samples of SCRFD subgroup, caries was removed up to firm dentin layer using a sharp excavator at pulpal cavity surface and up to hard dentin at periphery of the lesion based on the tissue resistance. Thus, the preparation of Class II cavities for all samples included in the study was completed. Subsequently, all the samples were restored using the restorative material of the group to which it was assigned as descripted below (Table 1). All the restorative materials were applied in accordance with the manufacturer’s instructions.

**Table 1:** Material type and compositions of the restorative materials used in this study.

Study group	Material type	Commercially brand name	Composition	Manufacturer
Group 1	Glass hybrid restorative material	Equia Forte® HT	Powder: fluoroaluminosilicate glass, polyacrylic acid, iron oxide Liquid: polybasic carboxylic acid, water	GC Corporation, Tokyo, Japan
Group 2	Conventional glass ionomer restorative material	Ionofil Molar	Water, pure polyacrylic acid, tartaric acid, aluminofluorosilicate glass and pigments	Voco, Cuxhaven, Germany
Group 3	Polyacid-modified composite resin	Dyract XP	UDMA Strontium-fluoro-silicate glass, strontium fluoride, TCB resin, photoinitiator and stabilizers	Dentsply, DeTrey, Konstanz, Germany

### Restoration procedures

**Group 1—Glass hybrid restorative (GHR) (Equia Forte**^**®**^ **HT):** After the cavity preparation by using SCRFD or CCRSD techniques, GC cavity conditioner (containing mild polyacrylic acid solution, GC America Inc.) was applied to the cavity surfaces for 10 s and rinsed and gently dried. Then, the capsule of Equia Forte^®^ HT system (GC America Inc., Alsip, IL, USA) was mixed for 10 s. Subsequently, restorative material was applied to all the cavities and contoured. After finishing the restoration, Equia Forte^®^ Coat (GC America Inc., Alsip, IL, USA) was applied and light cured for 20 s.

**Group 2—Conventional glass ionomer restorative (CGIR) (Ionofil Molar):** After the cavity preparation by using SCRFD or CCRSD techniques, the powder and liquid of the restorative material were mixed according to the manufacturer’s instructions and applied to the cavity. Subsequently, Final Varnish LC (Voco, Cuxhaven, Germany) was applied, and light cured for 10 s. After 7 min, the restoration was polished and Final Varnish LC was re-applied and light cured for 10 s.

**Group 3—Compomer (Dyract XP):** After the cavity preparation by using SCRFD or CCRSD techniques, adhesive material (Prime&Bond^®^ NT, Dentsply DeTrey GmbH, Konstanz, Germany) was applied to the cavity surfaces and light cured. Subsequently, Dyract XP (Dentsply DeTrey GmbH, Konstanz, Germany) was applied in increments up to a 3 mm. thickness immediately after the application of the adhesive. Restorative material was light cured for 20 s and polished with diamond burs.

The specimens were stored in distilled water at 37 °C after the restorative procedures. After the completion of the restorations, the teeth samples were thermocycled to mimic intraoral thermal changes for 10,000 cycles in water at 5 and 55 °C (SD Mechatronik GMBH, Feldkirchen-Westerham, Germany). After these procedures, the specimens were subjected to internal adaptation assessment using a Micro-CT device.

### Internal adaptation (IA) evaluation

**µCT scanning:** The samples were 3D scanned with a high-resolution desktop µCT system (Bruker microCT Systems 1275, Kontich, Belgium). The parameters used for the scanning were: 80 kVp, 125 mA, 1.0 mm Al filter, 26.7 µm pixel size, rotation at 0.2 steps. The mean duration of Micro-CT scanning was approximately 16 min.

**Micro-CT image reconstructions:** NRecon (ver. 1.7.4, Bruker Micro-CT Systems, Kontich, Belgium) software was used for the reconstruction. NRecon was used to create around 1,200 axial 2-dimensional images, which are 1,024 × 1,024 pixels with a 16-bit gray level. CTAn (v. 1.20.3 Bruker microCT Systems, Kontich, Belgium) software was used for the 3D image analysis. CTVox (v. 3.3.0 Bruker microCT Systems, Kontich, Belgium) was used for 3D visualization. 3D micro architecture of each specimen was analyzed with the selection of the region of interest (ROI) to detect the IA.

**Figure 2:**
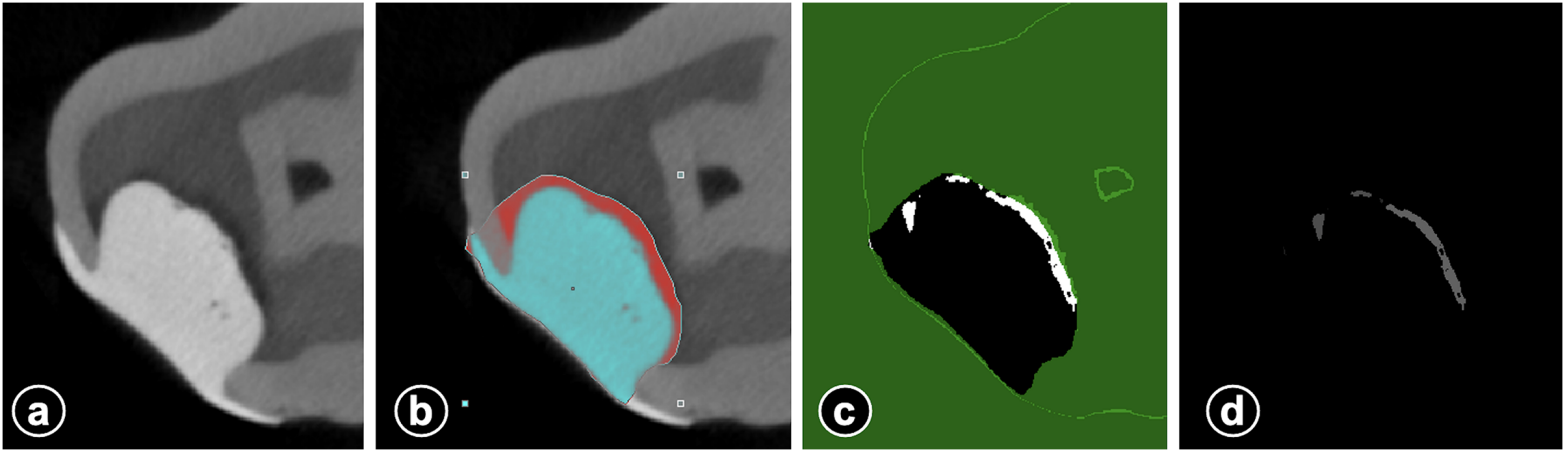
The figure series demonstrating the calculation of internal voids. Micro CT image of restorative material and voids (A) selection of ROI for determination of void volume (blue shows restorative material) (B) threshold segmentation of void and subtraction of restorative material (C) and void volume for automatic calculation (D).

**µCT evaluation:** CTAn software was used for the segmentation of the dentin, enamel, restorations, and void by using global thresholding. Thresholding is used to obtain only black/white pixels from the grayscale images. The black/white images used for the calculations for the detection of void volumes and restorations are calculated only from the ROI. The volumes obtained (mm^3^) for restorations and voids were used for the calculations of the percent of the void in the restorations (Fig. 2). Total cavity volume (mm^3^) and internal void volume (mm^3^) was calculated for each sample. Subsequently, the percentage (%) of internal void was calculated by proportioning the internal void volume to the total cavity volume. µCT evaluation-similar to caries removal and restorative procedures-was performed by a single operator (A.B.) with experience in this field. Micro-CT assessments were blindly performed by the same investigator (A.B.). The investigator was blinded to the sample origin.

### Fracture resistance (FR) evaluation

FR values of the restorations were measured using Lloyd universal testing machine (Lloyd Instruments, LRX, Ametek, West Sussex, UK). The stainless-steel bar (6 mm diameter) was placed perpendicularly into the center of the restoration at occlusal surface. The steel bar was adjusted to simultaneously contact all the occlusal surfaces of the restorations during the test period. The pressure was set as 1 mm/min of crosshead speed and the data were recorded in Newton unit at the time the fracture first occurred.

### Statistical analysis

To analyze the data, SPSS 11.5 software (SPSS, Inc., Chicago, IL, USA) was used. Mean ± standard deviation (SD) and median (min-max) were considered as descriptors for quantitative variables, and the number of samples (%) for qualitative variables. The data were analyzed by Student’s t, one-way ANOVA, Kruskal-Wallis H and Tukey *post hoc* tests. The correlation between IA and FR values was analyzed with Pearson test. Statistical significance level was considered as 5%.

## Results

**Figure 3:**
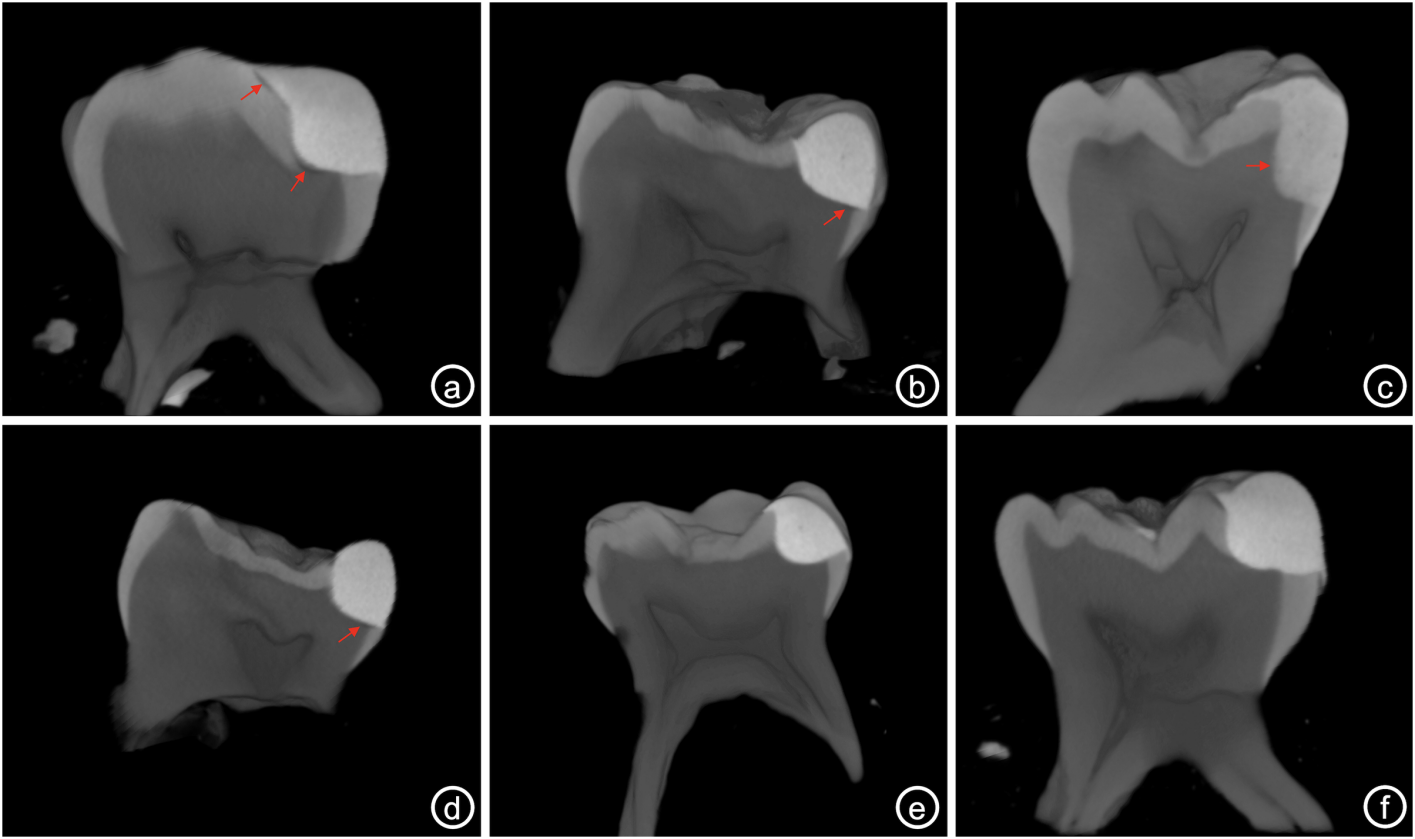
Figure series showing the study results for all subgroups. Representative Micro-CT images of cavities restored with diûerent restorative materials after CCRSD and SCRFD. Red arrows indicate the internal voids at the restorative material-dentin interface (A) CGIR applied after SCRFD, (B) GHR applied after SCRFD, (C) compomer applied after SCRFD, (D) CGIR applied after CCRSD, (E) GHR applied after CCRSD and (F) compomer applied after CCRSD.

For each restorative material, data were statistically analyzed whether there was a difference between caries removal techniques in terms of IA and FR values (Table 2). CCRSD technique showed statistically significantly superior IA results than SCRFD in all restorative materials (Table 2, Fig. 3) (*p* < 0.001 for GHR, *p* = 0.002 for CGIR and *p* < 0.001 for compomer). On the other hand, no statistical difference was found between the caries removal techniques in terms of FR in all restorative materials (Table 2, Fig. 3) (*p* = 0.548 for GHR, *p* = 0.163 for CGIR and *p* = 0.701 for compomer).

**Table 2:** The statistical comparisons of caries removal techniques for all the restorative materials.

Restorative materials	Caries removal technique	Internal voids (%)			Fracture resistance (Newton)		
		Mean ± SD	Median (Min-Max)	*p* value	Mean ± SD	Median (Min-Max)	*p* value
Glass hybrid restorative (Equia Forte HT)	CCRSD	2.43 ± 0.21	2.35 (2.20–2.70)	<0.001^a^	371.40 ± 12.86	371.00 (356.00–387.00)	0.548^a^
	SCRFD	3.61 ± 0.30	3.71 (3.11–3.90)		365.00 ± 18.85	365.00 (344.00–388.00)	
Conventional glass ionomer restorative (Ionofil Molar)	CCRSD	2.62 ± 0.21	2.58 (2.31–2.83)	0.002^a^	325.40 ± 10.97	329.00 (311.00–339.00)	0.163^a^
	SCRFD	3.68 ± 0.40	3.91 (3.23–4.00)		315.60 ± 9.10	316.00 (301.00–325.00)	
Compomer (Dyract XP)	CCRSD	1.84 ± 0.13	1.91 (1.63–1.96)	<0.001^a^	440.00 ± 12.51	439.00 (429.00–460.00)	0.701^a^
	SCRFD	3.40 ± 0.25	3.40 (3.10–3.70)		444.40 ± 21.27	449.00 (412.00–469.00)	

Notes:

a Student-t Test.

SD, Standart Deviation; Min, Minimum; Max, Maximum.

For each caries removal technique, data were statistically analyzed whether there was a difference between restorative materials in terms of IA and FR (Table 3, Fig. 3). Accordingly, it was found that statistically significant difference between restorative materials in terms of IA and FR in CCRSD (Table 3) (*p* < 0.001 and *p* < 0.001, respectively). In the statistical comparison of IA results, Tukey *post hoc* analysis was used to determine between which restorative material groups the statistical difference was significant. According to the binary comparisons, while compomer showed statistically superior IA results than both GHR and CGIR materials (*p* = 0.001 and *p* < 0.001, respectively), no statistically significant difference was determined between GHR and CGIR materials. In the statistical comparison of FR results, Tukey *post hoc* analysis was used to determine between which restorative material groups the statistical difference was significant. According the binary comparisons, compomer showed statistically superior FR results than both GHR and CGIR materials (*p* < 0.001 and *p* < 0.001, respectively). Additionally, GHR was statistically more resistant to fracture than CGIR (*p* < 0.001) (Table 3, Fig. 3).

**Table 3:** The statistical comparisons of restorative materials for caries removal techniques.

Caries removal technique	Restorative materials	Internal voids (%)			Fracture resistance (Newton)		
		Mean ± SD	Median (Min-Max)	*p* value	Mean ± SD	Median (Min-Max)	*p* value
CCRSD	Glass hybrid restorative (Equia Forte HT)	2.43 ± 0.21	2.35 (2.20–2.70)	<0.001^a^	371.40 ± 12.86	371.00 (356.00–387.00)	<0.001^a^
	Conventional glass ionomer restorative (Ionofil Molar)	2.62 ± 0.21	2.58 (2.31–2.83)		325.40 ± 10.97	329.00 (311.00–339.00)	
	Compomer (Dyract XP)	1.84 ± 0.13	1.91 (1.63–1.96)		440.00 ± 12.51	439.00 (429.00–460.00)	
SCRFD	Glass hybrid restorative (Equia Forte HT)	3.61 ± 0.30	3.71 (3.11–3.90)	0.212^b^	365.00 ± 18.85	365.00 (344.00–388.00)	<0.001^a^
	Conventional glass ionomer restorative (Ionofil Molar)	3.68 ± 0.40	3.91 (3.23–4.00)		315.60 ± 9.10	316.00 (301.00–325.00)	
	Compomer (Dyract XP)	3.40 ± 0.25	3.40 (3.10–3.70)		444.40 ± 21.27	449.00 (412.00–469.00)	

Notes:

a One-Way ANOVA test.

b Kruskal Wallis H test.

SD, Standart Deviation; Min, Minimum; Max, Maximum.

In SCRFD technique, while it was found a statistically significant difference between the restorative materials for FR (*p* < 0.001), there was no difference for IA results (*p* = 0.212) (Table 3, Fig. 3). In the statistical comparison of FR results, Tukey *post hoc* analysis was used to determine between which restorative material groups the statistical difference was significant. According to the binary comparisons, compomer showed statistically superior FR results than both GHR and CGIR materials (*p* < 0.001 and *p* < 0.001, respectively). Additionally, GHR was statistically more resistant to fracture than CGIR (*p* = 0.002) (Table 3, Fig. 3).

**Figure 4:**
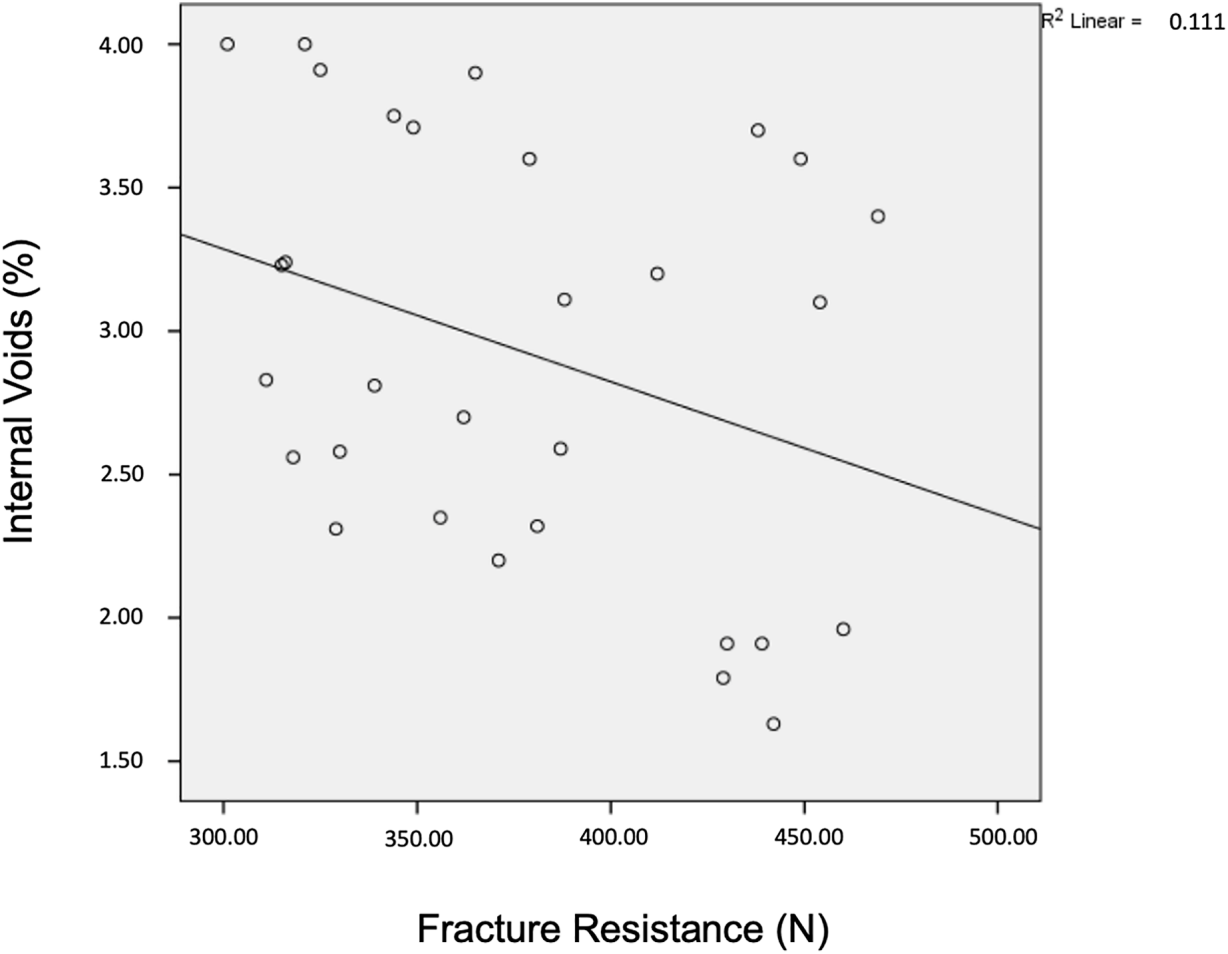
The correlation between IA and FR results. The table shows the correlation results of IA and FR. Accordingly, as the internal voids increase, the fracture resistance values decrease.

The correlation between internal void (%) and FR (N) values for all samples was also analyzed independent of the restorative materials. There was a moderate negative correlation between the two mentioned variables, however this correlation was not statistically significant (r = −0.333, *p* = 0.072). As the internal voids increase (decrease in IA), FR values decrease and the graph of this correlation is given in Fig. 4.

## Discussion

The present study evaluated the IA and FR of resin and glass ionomer based restorative materials applied after conventional or selective caries removal procedures *in-vitro*.

The cavitated caries lesions are still an ongoing oral health problem, and the prevalence of caries in primary dentition reaches up to 90%. Since primary teeth are vital for the development of the child, it is necessary to keep these teeth in the mouth as much as possible (Rodrigues et al., 2019). Untreated caries lesions negatively affect the oral health related quality of life of both children and their parents (Rodrigues et al., 2019; Fernandes et al., 2017). Therefore, restorative treatments and materials are quite important and their success is critical for the effort of keeping primary teeth (Rodrigues et al., 2019). For this purpose; compomers, conventional glass ionomer cements and high viscosity/glass hybrid glass ionomer cements are among the most frequently used restorative materials (Shetty & Shetty, 2012; Dhar et al., 2015; Kisby, 2021). Compomers show better physical and bonding properties of glass ionomers, with the high esthetics of composites, ease of placement and good handling properties (Shetty & Shetty, 2012). Based on this information, compomer, conventional glass ionomer and glass hybrid restorative were included in the study design due to the being the most commonly used restorative materials in pediatric dentistry.

The management of the carious lesions has been wide-ranging in primary teeth. Although the complete removal of the caries is one of the primary approaches, today, selective caries removal procedures are among the contemporary approaches (Innes et al., 2016; Schwendicke et al., 2016; Kher & Rao, 2019). The extent of carious tissue removal is closely related to the hardness level of the remaining dentin tissue. Clinically, subjectively determinable hardness levels of the remaining dentin are listed as soft, leathery, firm and hard. Evaluating the force required for a sharp dental explorer to perform a mark on carious dentin is the most convenient method for the operator to make a decision the degree of hardness of carious dentin (Ricketts et al., 2013; Kher & Rao, 2019). In moderately deep lesions, selective caries removal to firm dentin is recommended to an adequate depth that leaves the base of the lesion in firm dentin (Kher & Rao, 2019). Firm dentin is resistant to hand excavation requiring some pressure to be applied through an instrument to remove it. However, the caries in the peripheral areas of the lesion should be removed to hard dentin to provide peripheral seal zone (Innes et al., 2016; Schwendicke et al., 2016; Kher & Rao, 2019). Therefore, it is important to design new research on selective caries removal procedures to be applied before the application of restorative materials used in pediatric dentistry. Thus, it may be possible to investigate the effects of selective caries removal on different properties (such as internal adaptation, fracture resistance) of restorations and to make specific conclusions. Therefore, selective caries removal procedures were included in the study design and methodology.

IA and FR tests are frequently used to test the properties of restorative materials under *in-vitro* conditions (Virmani, Tandon & Rao, 1997; Murray et al., 2002; Seraj et al., 2015; Yildiz, Simsek & Pamir, 2016; Al Tuwirqi et al., 2021; Oguz et al., 2021). In light of the mentioned information, it was aimed to evaluate the IA and FR of resin and glass ionomer based restorative materials applied after CCRSD and SCRFD. Internal adaptation investigations have been carried out using dye penetration, optical coherence tomography, scanning electron microscopy and Micro-CT methods. Although each mentioned method has some advantages, Micro-CT imaging method was used in the presented study because it is non-invasive nature and allows 3D examination and internal voids were presented as % by proportioning the total cavity volume in 3D (Abdelaziz et al., 2020; Al Tuwirqi et al., 2021; Demirel et al., 2021). In this regard, this study has some limitations. Carious primary molar teeth were used since it was planned to remove the caries to firm dentin in SCRFD technique. Since the firm dentin does not present in the caries obtained artificially, caries-free primary teeth were not preferred. Instead of this, the primary teeth with dentinal carious in the depth of moderate level were used. Therefore, the cavities of all the samples in this study were not standardized dimensions and there were minor differences. To tolerate mentioned limitation, internal voids were recorded as % and analyzed to allow a more accurate comparison. Also, to date, limited information is available in the literature that provide results about the relation between IA and FR values. Thus, the determination of the correlation between IA and FR is the secondary objective of this research and the results will help to achieve information regarding the effect of IA on FR.

There are very few studies (Shetty & Shetty, 2012; Al Tuwirqi et al., 2021) in dentistry literature evaluating the internal adaptation quality of restorative materials used in pediatric dentistry, and limited information is available on this topic. Since compomers are resin-containing materials, attention should be focused on polymerization shrinkage (Shetty & Shetty, 2012). If the polymerization shrinkage is not within the acceptable limits, the adaptation of restorative material to dental tissues become suspicious and internal voids occur between the material and tooth structures. These can cause secondary caries, discoloration or postoperative sensitivity and pulpal inflammation (Murray et al., 2002; Shetty & Shetty, 2012). Therefore, it is important to evaluate IA of restorative materials. According to the findings of the presented research, in the use of GHR, CGIR and compomer materials, CCRSD showed superior internal adaptation results than SCRFD with statistically significant difference. Also, compomer showed superior IA results than both GHR and CGIR with statistical significance in CCRSD. On the other hand, there was no statistical difference between all the restorative materials regarding IA results in SCRFD.

One study comparatively evaluated the marginal adaptations of compomer, composite and glass ionomers after conventional cavity preparation by using SEM method (Gjorgievska et al., 2008). The authors reported that the composite and compomer showed superior adaptability than glass ionomers. Similarly, in this study, in CCRSD technique, compomer material showed superior IA results than GHR and CGIR. Glass ionomer materials are self-adhesive restoratives that does not require any bonding material. The adhesion mechanism of glass ionomers is related to the formation of ionic adhesion with mineralized tooth tissues. The lack of IA in glass ionomers might occur due to the absence of an adhesive and the shorter working time after mixing to insert the high viscosity glass ionomer into the cavity (Mendonça et al., 2021). In one study, authors reported that high viscosity glass ionomer material (Equia Forte Fil) showed more internal gap formation with statistical significance than light-cured composites in permanent teeth (Mendonça et al., 2021). On the other hand, GHR adapted to the cavity walls better than CGIR without statistically significant difference in CCRSD in this study. Conditioning the dental surfaces (such as dentin) before the placement of glass ionomer restoratives may restrict the development of ion-exchange layer. Also, the pre-treatment of cavity surfaces with a conditioner with polyacrylic acid and aluminum chloride is recommended to provide the smear layer removal. Otherwise, leaving the smear layer hinders the formation of an adhesive layer between tooth surfaces and glass ionomers (Gjorgievska et al., 2008; Mendonça et al., 2021). In the present study, the cavity conditioner was used before GHR placement to remove smear layer and it can be thought that cavity conditioning was an important factor relating to the better adaptation results of GHR than CGIR. On the other hand, there was no statistically significant difference between all the restorative materials in terms of IA in SCRFD. Based on the above-mentioned findings, it can be concluded that the use of resin-containing materials in restorative procedures performed with conventional cavity preparation will enhance IA quality. In addition, it is possible to say that approaches that prevent the lack of IA will be beneficial in selective caries removal independent of the restorative material.

In FR evaluation, there was no statistical difference between CCRSD and SCRFD techniques in restoration of GHR, CGIR and compomer materials. On the other hand, statistically significant differences were found between the restorative materials in terms of FR in CCRSD and SCRFD technique. According to these results, compomer was more resistant to load to fracture with statistically significant difference than both GHR and CGIR. In addition, GHR showed statistically superior FR than CGIR. Similar to the present study, a previous study reported that resin-based restorative materials such as compomers and composites had statistically higher FR than glass-ionomer-containing materials in primary molars (Yildiz, Simsek & Pamir, 2016). Restoration fracture was reported as the main cause for the failure of CGIR restorations in class II cavities. This mode of fracture is attributed to insufficient physical properties of glass ionomer restoratives (Qvist et al., 2004). In this study, the failure of glass ionomers under fracture loads was attributed to the mentioned situation. On the other hand, glass hybrid glass ionomers showed superior mechanical results under fracture loads than conventional glass ionomer restoratives. One study stated that Fuji XI, a high-viscosity glass ionomer, was superior to Fuji II LC and Vitremer in terms of cuspal fracture strength. In this study, the superior resistance of GHR to fracture load compared to CGIR was attributed to its reinforced mechanical properties (Virmani, Tandon & Rao, 1997). In this context, based on the results of the present study, although the use of resin-containing materials provided high FR, if glass ionomers are to be used due to the other advantages they offer, the use of glass hybrid glass ionomers should be recommended in both conventional and selective caries removal procedures.

Although IA and FR have been the subject of many studies in the dental literature, very limited information is available on the correlation between them, especially in the restorative procedures of primary teeth. In this study, IA evaluation was completed as the first step of the research and then FR test was performed. In this context, both IA and FR data were recorded for each tooth sample. According to the correlation test of IA and FR results, a medium negative correlation was found between the internal voids and FR values. Although the result did not present a statistically significant difference, FR of the restoration decreased as the internal voids increased (IA decreased). For this reason, it is possible to say that all interventions to strengthen the IA during the application of restorations will contribute to increasing the FR of the restorations.

This study has some limitations and strengths. One of the main limitations was that the cavities and restorations were not standard sizes, as carious teeth were used, as abovementioned in detail. On the other hand, this limitation also poses a standardization problem for FR assessment. However, the presence of carious tissue in tooth samples and the inclusion of specific characteristics of the caries constitute the strength of this study. Another limitation is that this work was performed under *in-vitro* conditions. This creates limitations due to the elimination of intraoral environment variables. In order to reduce this limitation to some extent, subjecting the tooth samples to *in vitro* tests after artificial aging or chewing simulation may be the subject of further studies.

## Conclusions

Within the limitations of the method design, the following conclusions could be drawn from this *in-vitro* study:

- Conventional caries removal showed superior internal adaptation results than selective caries removal independent of the restorative material. In this regard, when selective removal is preferred, it should be ensured that caries removal is performed at least up to the hard dentin at the periphery of the lesion.
- In conventional caries removal, compomer showed superior internal adaptation and fracture resistance results than glass ionomers. Also, glass hybrid restoratives were more resistant to fracture than the conventional glass ionomers.
- When selective caries removal was preferred, no statistical difference was found between the restorative materials in terms of internal adaptation. In fracture resistance evaluation, compomer was found to be the most resistant material followed by glass hybrid and conventional glass ionomers. Compomers are recommended when selective caries removal is preferred. On the other hand, if indications point to the use of glass ionomers, the use of glass hybrid restoratives should be recommended due to their higher resistance to fracture.
- Since the increase in the internal adaptation correlates with the increase in the fracture resistance, it is possible to conclude that the adaptation quality will increase the fracture resistance of the restoration. Therefore, clinicians should give importance to interventions to increase the internal adaptation quality of the restorations.

## Supplemental Information

10.7717/peerj.14825/supp-1 Supplemental Information 1 Raw Data.

## Additional Information and Declarations

### Data Availability

The following information was supplied regarding data availability: The raw data is available as a Supplemental File.

## References

[ref-1] Abdelaziz M, Zuluaga AF, Betancourt F, Fried D, Krejci I, Bortolotto T (2020). Optical coherence tomography (OCT) for the evaluation of internal adaptation of class V resin restorations on dentin. Proceedings of SPIE—The International Society for Optical Engineering.

[ref-2] Al Tuwirqi AA, El Ashiry EA, Alzahrani AY, Bamashmous N, Bakhsh TA (2021). Tomographic evaluation of the internal adaptation for recent calcium silicate-based pulp capping materials in primary teeth. Biomed Research International.

[ref-3] Alani AH, Toh CG (1997). Detection of microleakage around dental restorations: a review. Operative Dentistry.

[ref-4] Bayrak GD, Sandalli N, Selvi-Kuvvetli S, Topcuoglu N, Kulekci G (2017). Effect of two different polishing systems on fluoride release, surface roughness and bacterial adhesion of newly developed restorative materials. Journal of Esthetic and Restorative Dentistry.

[ref-5] Brkanović S, Ivanišević A, Miletić I, Mezdić D, Jukić Krmek S (2021). Effect of nano-filled protective coating and different pH enviroment on wear resistance of new glass hybrid restorative material. Materials.

[ref-6] Chadwick BL, Evans DJ (2007). Restoration of class II cavities in primary molar teeth with conventional and resin modified glass ionomer cements: a systematic review of the literature. European Archives of Paediatric Dentistry.

[ref-7] Demirel G, Orhan AI, Irmak O, Aydın F, Büyüksungur A, Bilecenoğlu B, Orhan K (2021). Effects of preheating and sonic delivery techniques on the internal adaptation of bulk-fill resin composites. Operative Dentistry.

[ref-8] Dhar V, Hsu KL, Coll JA, Ginsberg E, Ball BM, Chhibber S, Johnson M, Kim M, Modaresi N, Tinanoff N (2015). Evidence-based update of pediatric dental restorative procedures: dental materials. Journal of Clinical Pediatric Dentistry.

[ref-9] Ehlers V, Gran K, Callaway A, Azrak B, Ernst CP (2019). One-year clinical performance of flowable bulk-fill composite vs conventional compomer restorations in primary molars. The Journal of Adhesive Dentistry.

[ref-10] Fernandes IB, Costa DC, Coelho VS, Sá-Pinto AC, Ramos-Jorge J, Ramos-Jorge ML (2017). Association between sense of coherence and oral health-related quality of life among toddlers. Community Dental Health.

[ref-11] Finucane D (2012). Rationale for restoration of carious primary teeth: a review. European Archives of Paediatric Dentistry.

[ref-12] Finucane D (2019). Restorative treatment of primary teeth: an evidence-based narrative review. Australian Dental Journal.

[ref-13] Frencken JE, Peters MC, Manton DJ, Leal SC, Gordan VV, Eden E (2012). Minimal intervention dentistry for managing dental caries—A review: report of a FDI task group. International Dental Journal.

[ref-14] Gjorgievska E, Nicholson JW, Iljovska S, Slipper IJ (2008). Marginal adaptation and performance of bioactive dental restorative materials in deciduous and young permanent teeth. Journal of Applied Oral Science.

[ref-15] Han SH, Park SH (2017). Comparison of internal adaptation in class II bulk-fill composite restorations using micro-CT. Operative Dentistry.

[ref-16] Innes NP, Frencken JE, Bjørndal L, Maltz M, Manton DJ, Ricketts D, Van Landuyt K, Banerjee A, Campus G, Doméjean S, Fontana M, Leal S, Lo E, Machiulskiene V, Schulte A, Splieth C, Zandona A, Schwendicke F (2016). Managing carious lesions: consensus recommendations on terminology. Advances in Dental Research.

[ref-17] Kher MS, Rao A, Kher MS, Rao A (2019). Lesion management: selective removal of carious tissue in shallow, moderately deep, and deep carious lesions. Contemporary Treatment Techniques in Pediatric Dentistry.

[ref-18] Kim HJ, Park SH (2014). Measurement of the internal adaptation of resin composites using micro-CT and its correlation with polymerization shrinkage. Operative Dentistry.

[ref-19] Kisby L (2021). Glass-hybrid restorations in pediatric patients. Compendium Continuing Education in Dentistry.

[ref-20] Krithikadatta J, Gopikrishna V, Datta M (2014). CRIS guidelines (checklist for reporting in-vitro studies): a concept note on the need for standardized guidelines for improving quality and transparency in reporting in-vitro studies in experimental dental research. Journal of Conservative Dentistry.

[ref-21] Krämer N, Frankenberger R (2007). Compomers in restorative therapy of children: a literature review. International Journal of Paediatric Dentistry.

[ref-22] Mendonça BC, Romano BC, Sebold M, Fronza BM, Braga RR, Nima G, Price RB, Giannini M (2021). Polymerization shrinkage stress, internal adaptation, and dentin bond strength of bulk-fill restorative materials. International Journal of Adhesion and Adhesives.

[ref-23] Murray PE, Hafez AA, Smith AJ, Cox CF (2002). Bacterial microleakage and pulp inflammation associated with various restorative materials. Dental Materials.

[ref-24] Ngo HC, Mount G, Mc Intyre J, Tuisuva J, Von Doussa RJ (2006). Chemical exchange between glass-ionomer restorations and residual carious dentine in permanent molars: an in vivo study. Journal of Dentistry.

[ref-25] Oguz EI, Bezgin T, Orhan AI, Orhan K (2021). Comparative evaluation of adaptation of esthetic prefabricated fiberglass and CAD/CAM crowns for primary teeth: microcomputed tomography analysis. Biomed Research International.

[ref-26] Qvist V, Laurberg L, Poulsen A, Teglers PT (2004). Class II restorations in primary teeth: 7-year study on three resin-modified glass ionomer cements and a compomer. European Journal of Oral Sciences.

[ref-27] Rengo C, Goracci C, Ametrano G, Chieffi N, Spagnuolo G, Rengo S, Ferrari M (2015). Marginal leakage of class V composite restorations assessed using microcomputed tomography and scanning electron microscope. Operative Dentistry.

[ref-28] Ricketts D, Lamont T, Innes NP, Kidd E, Clarkson JE (2013). Operative caries management in adults and children. Cochrane Database of Systematic Reviews.

[ref-29] Rodrigues JA, Casagrande L, Araújo FB, Lenzi TL, Mariath AAS, Leal SC, Takeshita EM (2019). Restorative materials in pediatric dentistry, pediatric restorative dentistry. Pediatric Restorative Dentistry.

[ref-37] Šalinović I, Stunja M, Schauperl Z, Verzak Ž, Ivanišević Malčić A, Brzović Rajić V (2019). Mechanical properties of high viscosity glass ionomer and glass hybrid restorative materials. Acta Stomatologica Croatica.

[ref-30] Schwendicke F, Frencken JE, Bjørndal L, Maltz M, Manton DJ, Ricketts D, Van Landuyt K, Banerjee A, Campus G, Doméjean S, Fontana M, Leal S, Lo E, Machiulskiene V, Schulte A, Splieth C, Zandona AF, Innes NP (2016). Managing carious lesions: consensus recommendations on carious tissue removal. Advances in Dental Research.

[ref-31] Seraj B, Ghadimi S, Estaki Z, Fatemi M (2015). Fracture resistance of three different posts in restoration of severely damaged primary anterior teeth: an in vitro study. Dental Research Journal (Isfahan).

[ref-32] Shetty SM, Shetty RG (2012). Adaptation of different compomers to primary teeth cavities. Journal of Orofacial Research.

[ref-33] Uchimura JYT, Sato F, Santana RG, Menezes-Silva R, Bueno LS, Borges AFS, de Lima Navarro MF, Nicholson JW, Sidhu SK, Pascotto RC (2021). Translucency parameter of conventional restorative glass-ionomer cements. Journal of Esthetic and Restorative Dentistry.

[ref-34] Virmani S, Tandon S, Rao N (1997). Cuspal fracture resistance and microleakage of glass ionomer cements in primary molars. Journal of Clinical Pediatric Dentistry.

[ref-35] Walsh LJ, Brostek AM (2013). Minimum intervention dentistry principles and objectives. Australian Dental Journal.

[ref-36] Yildiz E, Simsek M, Pamir Z (2016). Fracture strength of restorations in proximal cavities of primary molars. Scanning.

